# Perceiving Change: Local Perspectives on Ecological Transformation and Sustainability Dynamics in the Alpine Lake Idro Ecosystem

**DOI:** 10.1007/s00267-026-02553-7

**Published:** 2026-07-07

**Authors:** Chiara Mazzocchi, Simone Manna

**Affiliations:** Department of Agricultural and Environmental Science, Production, Territory, Agroenergy, University of Milan, Milan, Italy

**Keywords:** Alpine lake, Stakeholder perceptions, Qualitative analysis, Water governance, Socio-ecological systems, Sustainable territorial development

## Abstract

Freshwater lakes are increasingly affected by interacting ecological, infrastructural and socio-economic pressures, yet research on regulated deep lake systems has mainly focused on biophysical processes, with comparatively limited attention to how local stakeholders perceive ecological change and sustainability transitions. This study addresses this gap by examining how stakeholders interpret environmental change, water governance and future sustainability in Lake Idro, a regulated deep subalpine lake in northern Italy. The novelty of the paper lies in integrating limnological evidence with qualitative stakeholder perspectives to show how perceived ecological improvement, governance conflicts and institutional trust jointly shape local understandings of sustainability. The study combines documentary and scientific evidence with a focus group and semi-structured interviews with 21 stakeholders, including residents, institutional actors, environmental associations and tourism operators. Data were analyzed through an inductive approach inspired by the Gioia methodology. The findings identify two interrelated dimensions: Ecological Perception and Participatory Awareness, which captures how visible environmental changes and lived experience inform local interpretations of lake conditions; Territorial Governance and Local Development, which reflects concerns over water regulation, tourism, infrastructure and institutional legitimacy. Stakeholders generally perceive an improvement in the lake’s ecological and esthetic condition following reduced water-level fluctuations, while expressing concern that new hydraulic interventions could reintroduce ecological instability and undermine tourism-based development. By connecting ecological evidence with socially mediated perceptions of change, the study contributes to environmental management and adaptive water governance debates, highlighting the importance of knowledge exchange, participatory decision-making and institutional trust in managing regulated lake systems.

## Introduction

Freshwater lakes are fundamental components of natural landscapes, providing critical ecosystem services such as water supply, wildlife habitat, recreation and economic support for surrounding communities (Tockner 2021; Sterner et al. 2020). In recent years, growing attention has been devoted to understanding how diverse communities perceive environmental risks, ecosystem transformations and management trade-offs, particularly in relation to freshwater and terrestrial ecosystems (McDougall et al. 2020). For instance, Pachoud et al. (2020) analyzed how local stakeholders and tourists evaluated the ecosystem services of Alpine summer farms. Their results revealed a clear divergence of emphasis: stakeholders prioritized provisioning services linked to land-based livelihoods, whereas tourists emphasized cultural and regulating services, underscoring the influence of social roles and expectations in shaping perceptions of landscape value. Similarly, studies in southern Italy have shown that cultural, social, and economic contexts significantly affect how communities interpret environmental risks and which policy responses they view as legitimate or effective. Awareness of the ecosystem-services concept itself was associated with higher perceived value. Maran et al. (2023) further explored the evolving relationship between water resource exploitation and environmental awareness in the Alps. Drawing on archival research and oral histories, they traced how concerns over water quality, availability and ecosystem health gained recognition over time. Their analysis illustrated how stakeholder priorities shifted alongside growing environmental awareness and how institutional responsibilities gradually incorporated ecological criteria, reflecting a broader movement toward accountable and ecologically informed water governance. Comparable findings have emerged globally. Studies from Bangladesh, for example, show that communities along the Old Brahmaputra River express acute concern over pollution caused by solid waste, sewage discharge, and steamer traffic, linking these pressures to declines in biodiversity, fisheries, and public health (Islam and Chowdhury 2021). In Serbia’s Drina River Basin, residents’ perceptions of water quality often aligned closely with empirical measurements, especially when pollution was visible (Brankov et al. 2021). Conversely, in Peru’s Lake Junín region, community perceptions of pollution diverged from formal environmental indicators, as residents viewed the lake as degraded despite measured water quality meeting official standards (del Sante et al. 2025). Beyond lake systems, perceptions also shape evaluations of trade-offs in natural contexts. In Italy, local views of agroforestry systems highlight their environmental benefits while acknowledging economic and managerial challenges (Camilli et al. 2018). Similar dynamics were documented in Mexico, where cognitive-mapping exercises revealed how farmers and civil society actors conceptualize soil degradation drivers differently across crops and social groups (Arroyo-Lambaer et al. 2021). Collectively, these studies demonstrate that sustainability is not only an ecological or technical matter but also a social one. Understanding how stakeholders perceive ecosystem services, environmental risks and management policies is essential for designing inclusive, context-sensitive sustainability strategies. For freshwater lakes, where ecological processes intertwine with economic livelihoods and cultural identity, these insights are particularly relevant and Lake Idro, in northern Italy, exemplifies these dynamics. This deep, subalpine lake exhibits pronounced meromixis and has been the subject of extensive biogeochemical research in recent years (Tartari et al. 2021; Viaroli et al. 2018). While grounded in the specific context of Lake Idro, this study addresses broader questions concerning how local perceptions of ecological change interact with governance arrangements and sustainability dynamics in regulated freshwater systems. Previous research has shown that socio-ecological systems are shaped not only by biophysical processes, but also by institutional structures, stakeholder interactions and locally embedded knowledge (Berkes and Folke 1998; Folke et al. 2005; Ostrom 2009). In water governance research, adaptive water management has been proposed as a response to uncertainty, complexity, and competing resource uses. Rather than relying on fixed management prescriptions, adaptive approaches emphasize learning from management outcomes, stakeholder participation, institutional flexibility, and the capacity to revise rules and practices over time. These principles are particularly relevant for regulated lake systems, where ecological processes, infrastructure, water withdrawals, tourism, and local expectations interact across multiple temporal and institutional scales (Pahl-Wostl et al. 2007; Medema et al. 2008; Huitema et al. 2009; Pahl-Wostl 2009).

This study aims to investigate how different stakeholder groups perceive and interpret the sustainability of the ecological and socio-economic evolution of Lake Idro and to explore the future development scenarios they envision for the system.

Building on the socio-ecological systems literature, which highlights the interplay between biophysical processes, institutional arrangements and local knowledge (Berkes and Folke 1998; Folke et al. 2005; Ostrom 2009), the paper examines how local perceptions of environmental change interact with governance dynamics in a regulated freshwater system.

The study contributes to existing research by integrating stakeholder perspectives with ecological evidence, showing how perceived environmental conditions, governance conflicts and levels of participatory awareness shape sustainability trajectories over time. In doing so, it responds to calls for a stronger integration of social science approaches in environmental management, demonstrating how perception-based methods can uncover socio-ecological feedbacks and management tensions that may remain hidden in purely biophysical analyses (Reed 2008; Bennett et al. 2016; Manna et al. 2026).

Conceptually, the paper advances an integrative perspective linking ecological perception, participatory awareness and territorial governance, offering a framework that can support comparative analysis across other alpine and subalpine lake systems facing competing water uses and multi-level governance challenges (Flyvbjerg 2006; Pahl-Wostl 2009). From a management perspective, the findings highlight the importance of locally grounded knowledge and stakeholder engagement in supporting more adaptive and inclusive water governance processes (Bennett et al. 2018).

## Methodology

### Methodological Approach

A qualitative research design was adopted to investigate how local stakeholders, including residents, institutions and associations, perceive environmental change in the Lake Idro ecosystem. This approach was selected to explore perceptions of anoxia, the condition of complete depletion of dissolved oxygen in water, leading the lack of support to aerobic aquatic organisms particularly in deeper layers of a lake and related territorial issues without seeking to establish causal relationships or quantify impacts. Instead, the study aimed to capture subjective experiences and situated understandings, grounded in participants lived realities. Quantitative or deterministic methods were deemed inadequate for mapping the complex and evolving web of relationships that characterize the lake’s socio-ecological system.

The inductive nature of qualitative inquiry allows insights to emerge directly from empirical data, guided by recurring patterns rather than predefined hypotheses. Data analysis followed the principles of grounded theory (Strauss and Corbin 1998), with particular reference to the Gioia methodology (Gioia et al. 2013). This inductive process facilitated the construction of theoretical categories that evolved dynamically from the data itself. By minimizing researcher preconceptions during data collection and analysis, the approach helped avoid omission or distortion of key themes (Thomas 2006). Furthermore, it illuminated how individuals interpret their surroundings, negotiate meaning, and mobilize contextual resources to construct identity and address challenges (Flint and Golicic 2009).

Data collection, analysis, and theory development proceeded iteratively until reaching theoretical saturation, the stage at which no new insights emerged from additional data. This cyclical process ensured that conceptual development was deeply grounded in empirical evidence, enhancing both validity and interpretive richness.

### Case Study Context

**Fig. 1:**
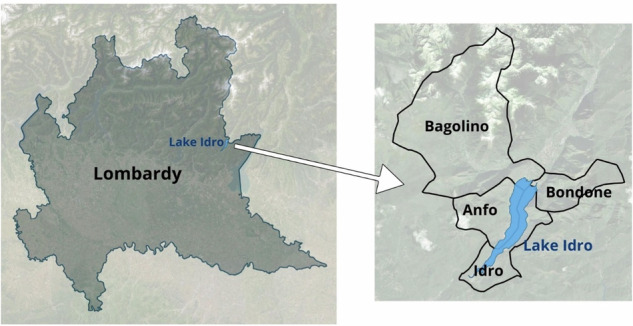
Map of Lake Idro and its localization in Lombardy on the right side; Lake Idro with all the municipalities bordering the lake, on the left side

Lake Idro is a glacial-origin lake located in the province of Brescia, in Lombardy region, near the Trentino border in northern Italy (Fig. 1). Situated at 368 m above sea level, it is both fed and drained by the Chiese River.

The lake covers ~10.9 km² and reaches a maximum depth of 122 m. It represents one of Europe’s earliest artificially regulated alpine lakes, and the first in Italy, to undergo formal hydraulic control. The concept of regulation dates back to 1855, when plans were developed to transform the lake into a reservoir supporting hydroelectric production and agricultural irrigation for the Brescian and Mantuan plains, which already held historical water rights.

The infrastructure concession included the construction of a drainage tunnel and a weir on the Chiese River. Initially, water levels fluctuated by up to 3.5 m, later extended to 7 m. Over time, the lake became a focal point for multiple, and often competing, uses, including irrigation, energy production, tourism, and environmental conservation. At the beginning of the 2000s, a coordinated regulatory system involving upstream reservoirs was introduced, reducing lake-level fluctuations from 7 to 3.25 m in an effort to balance economic use with ecological sustainability.

Since the late 1980s, Lake Idro has been known to experience hypolimnetic anoxia (Barbato 1990), primarily driven by artificial regulation, depth, and rising temperatures linked to climate change. Under natural conditions, vertical water mixing replenishes oxygen in deeper layers, sustaining aquatic life. However, reduced mixing in Lake Idro has led to persistent oxygen depletion at depth.

The lake was selected as a case study due to its long history of resource conflict and its relevance as a model for understanding tensions between economic development and environmental protection (Paris 2022). The study specifically aimed to assess how local stakeholders perceive the sustainability of the lake’s ecological and socio-economic evolution, and to explore the future scenarios they envision for the system’s development.

### Data Collection and Triangulation

**Fig. 2:**
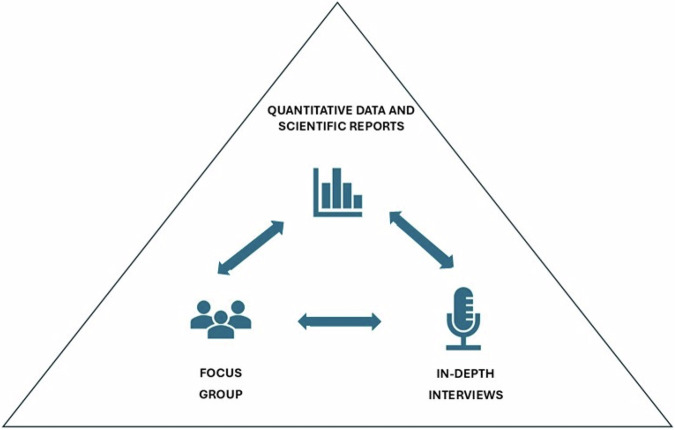
Triangulation of data sources

To ensure the robustness and credibility of findings, a triangulated data-collection strategy was employed (Fig. 2).

The methodology consisted of three sequential phases: (1) collection of data derived from the existing scientific literature and reports; (2) conducting a focus group; (3) carrying out in-depth, in-person interviews. The review of scientific literature and monitoring reports was conducted between January and March 2025. The exploratory focus group was held in March 2025, while semi-structured interviews were carried out between April and August 2025.

In the first phase, scientific literature and environmental monitoring data, including studies and regional reports, were reviewed to establish a factual baseline of Lake Idro’s hydrochemical and biological conditions. This review informed the refinement of the study’s central research question: “*Is the evolution of Lake Idro’s ecosystem perceived as sustainable? How do different stakeholder groups construct and interpret the sustainability of the Lake Idro ecosystem across environmental, social, and economic dimensions, and what key tensions emerge from their accounts?*”

The second phase involved a focus group with key local stakeholders, including municipal representatives (a mayor), two tourism operators (public and private), a local association representative, and two researchers. This session served two purposes: (i) to explore preliminary perceptions of the lake’s environmental condition and (ii) to identify relevant stakeholder categories for subsequent interviews. Insights from the focus group informed a purposive sampling strategy for the third phase, which consisted of semi-structured, in-person interviews. This method was chosen for its capacity to elicit detailed and reflective responses while allowing adaptive probing and contextual understanding (Knox and Burkard 2009). Interviews were conducted using a semi-structured format. The interview guide did not function as a fixed questionnaire, but as a set of thematic prompts used to orient the conversation toward the core research issue. This allowed the interviewer to maintain focus on the sustainability of the Lake Idro ecosystem while leaving space for participants to introduce their own meanings, concerns and priorities across environmental, social and economic dimensions. In this sense, the questions served as sensitizing prompts rather than as restrictive categories, consistent with an inductive, grounded-theory-informed approach. For this reason, first-order concepts emerged from the interaction between interviewer and interviewee, being shaped by the stimuli arising during the discussion rather than strictly derived from direct, pre-defined questions. Face-to-face interaction fostered trust and enabled observation of non-verbal cues, such as tone, gesture and setting, that enriched data interpretation. Compared with remote methods, in-person interviews enhanced ecological validity by situating participants within their lived environments. Integrating data from quantitative reports, focus group discussions and interviews enabled methodological triangulation, which strengthened reliability and provided a multi-perspective understanding of stakeholder perceptions. The resulting analysis yielded a grounded theoretical framework reflecting diverse experiential standpoints and local realities. Participants were selected purposively to reflect the range of institutional, economic and social perspectives within the Lake Idro area. The final sample comprised 21 individuals. The sample was categorized into three groups of stakeholders: (1) local community and civil society, (2) institutions and (3) companies. Local community representatives included citizens engaged in local associations working on social and environmental issues. Institutional actors referred to individuals holding formal roles within public administrations (e.g., municipality, mountain community, and regional authorities). Economic actors comprised entrepreneurs operating in the Lake Idro area, particularly in the tourism, hospitality and restaurant sectors (Table 1).

**Table 1:** Profiles of respondents

	Corporate/institutional role	Affiliation	Place	Role in the research
#1	Vice President	Environmental association 1	Municipality 1	Local community and civil society
#2	President	Environmental association 1	Municipality 1	Local community and civil society
#3	Ecologist	Independent researcher	Municipality 1	Institution
#4	Resident	Independent	Municipality 3	Local community and civil society
#5	Resident	Independent	Municipality 1	Local community and civil society
#6	Staff member	Camping 1	Municipality 1	Local community and civil society
#7	Mayor	Municipality 1	Municipality 1	Institution
#8	Mayor	Municipality 2	Municipality 2	Institution
#9	Civil Registry Officer	Municipality 1	Municipality 1	Institution
#10	Technical staff	Municipality 1	Municipality 1	Institution
#11	Mayor	Municipality 3	Municipality 3	Institution
#12	Seasonal worker	Camping 2	Municipality 3	Local community and civil society
#13	Owner	Camping 3	Municipality 4	Company
#14	Owner	Camping 4	Municipality 1	Company
#15	Manager	Camping 2	Municipality 3	Company
#16	HR Manager	Camping 5	Municipality 1	Company
#17	Owner	Vacation rentals 1	Municipality 1	Company
#18	Owner	Vacation rentals 2	Municipality 1	Company
#19	Owner	Camping 7	Municipality 1	Company
#20	Owner	Camping 1	Municipality 1	Company
#21	Owner	Bar 1	Municipality 3	Company

**Fig. 3:**
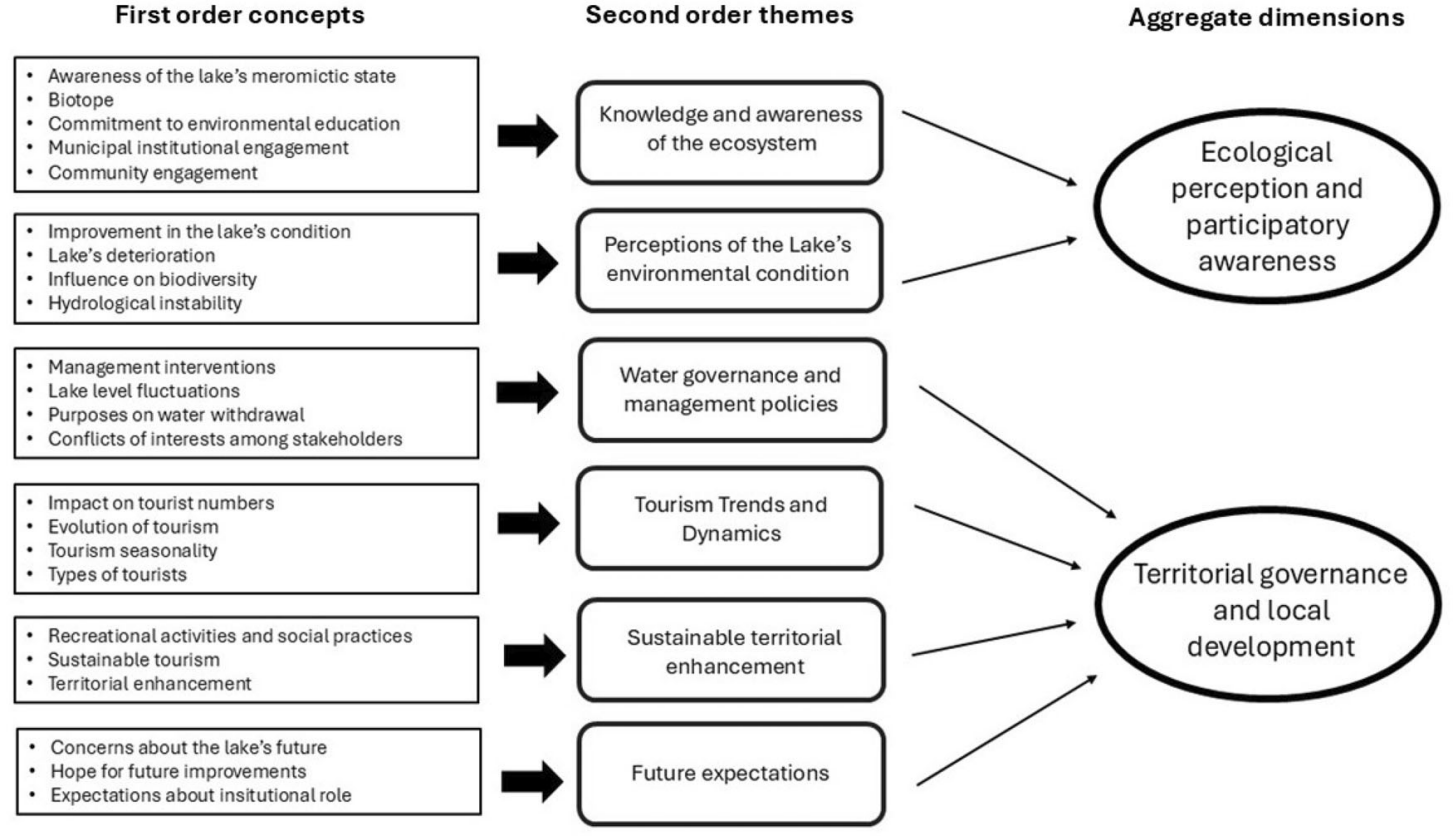
Framework of the qualitative analysis result

The third step of the research was addressed to the interviews, conducted in person following initial contact by e-mail or telephone. In fact, participant availability was previously coordinated through focus-group stakeholders, who assisted with identification and recruitment in March 2025. The interviews took place between April and August 2025, were recorded with informed consent and transcribed verbatim for rigorous qualitative analysis. Interviews were transcribed with the support of transcription software (Microsoft Word) and subsequently checked manually by the authors. An open-coding technique was applied, following the approach by Gioia et al. (2013), widely employed in literature (Grazia et al. 2024), by using Maxqda 24 software. The transcripts were independently coded by the authors, after which the initial coding results were consolidated. Through extensive discussion, we compared, merged, and refined our interpretations to ensure analytical consistency. Across several iterative cycles, the raw qualitative data were progressively aggregated, leading to the identification of 23 first-order concepts. These concepts were subsequently examined and synthesized through relevant theoretical lenses, producing six second-order themes. At this stage, the analysis advanced toward a higher level of theoretical abstraction, drawing on existing literature to identify emerging patterns that could enhance understanding of the phenomenon under investigation (Gioia et al. 2013). Finally, by analyzing the interrelationships among the second-order themes, two overarching aggregate dimensions were derived (Fig. 3, following paragraph).

## Results

The quantitative findings reported in scientific studies on Lake Idro’s anoxia (Viaroli et al. 2018; Tartari et al. 2021; Lombardy Region 2021) offer a factual basis for benchmarking and evaluating stakeholders’ perceptions of the lake’s condition.

Long-term observations indicate that Lake Idro has been meromictic at least since the late 1960s, with deep-water hypoxia below ~60 m and anoxia toward the bottom repeatedly documented in campaigns from 1969 to 1995 (Viaroli et al. 2018). The lake’s narrow, elongated basin and steep valley walls make it naturally prone to persistent density stratification. Solute-driven density differences reinforce thermal stratification, and external inputs of organic matter from the watershed, especially during periods of elevated trophic pressure, have progressively strengthened and stabilized the monimolimnion. Lake-level management and hydrology have also modulated surface-layer conditions. Periods of high withdrawal from the upper water column and large river inflows (linked to precipitation and melt dynamics) affected summer surface temperatures and promoted through-flow within the mixolimnion, without substantially altering the stability of deeper layers (Viaroli et al. 2018). Despite this strong stability, a rare full-lake mixing likely occurred during winter 2005–2006, similar to other deep perialpine systems in that anomalously cold period; however, the system subsequently returned to its stratified state. Idro’s present-day vertical structure features a gradual transition between the upper mixed waters and the deep, stably stratified monimolimnion rather than a razor-thin interface. This broad transition supports distinct microbial and biogeochemical regimes across depth (Tartari et al. 2021). Although background stability is high, a well-documented episode in August 2010 produced a surface discoloration that evolved over several weeks; this was attributed to partial entrainment from the upper deep layer during intense weather-hydrologic conditions, followed by rapid oxidation in the surface waters (Tartari et al. 2021). These events underscore that event-scale perturbations can transiently modify water-quality conditions even when full overturn is unlikely.

Because deep-water renewal is infrequent, the oxygen budget of the monimolimnion is constrained by limited vertical exchange and by ongoing mineralization of settling organic matter (Viaroli et al. 2018). Trophically, Lake Idro illustrates how meromixis can decouple surface conditions from whole-lake nutrient status. While volume-weighted metrics point to high trophic potential when the entire water column is considered, the mixolimnion often remains in the (oligo-)mesotrophic range because large nutrient pools are sequestered at depth. Community composition has shifted over decades, with cyanobacteria becoming more prominent from the late 1980s onward, and alternating dominance patterns consistent with interannual variability in nutrient availability and mixing intensity.

The findings of the qualitative interviews reveal multiple layers of perception (Fig. 3). These insights also illuminate the themes considered most relevant by the actors involved.

Two main aggregate dimensions emerged from our coding and interpretation process: (i) *Ecological Perception and Participatory Awareness* and (ii) *Territorial Governance and Local Development*. The first dimension encompasses what local actors know, perceive, and understand about the lake’s environmental dynamics, as well as the extent to which they feel engaged with its evolution. It thus provides an interpretative lens for understanding how individuals and groups perceive ecosystem transformations. The second dimension focuses on regulatory frameworks, political actions, water resource management, and the role of tourism as an economic driver, highlighting how institutions, the local economy, and tourism flows interact within the lake system.

**Fig. 4:**
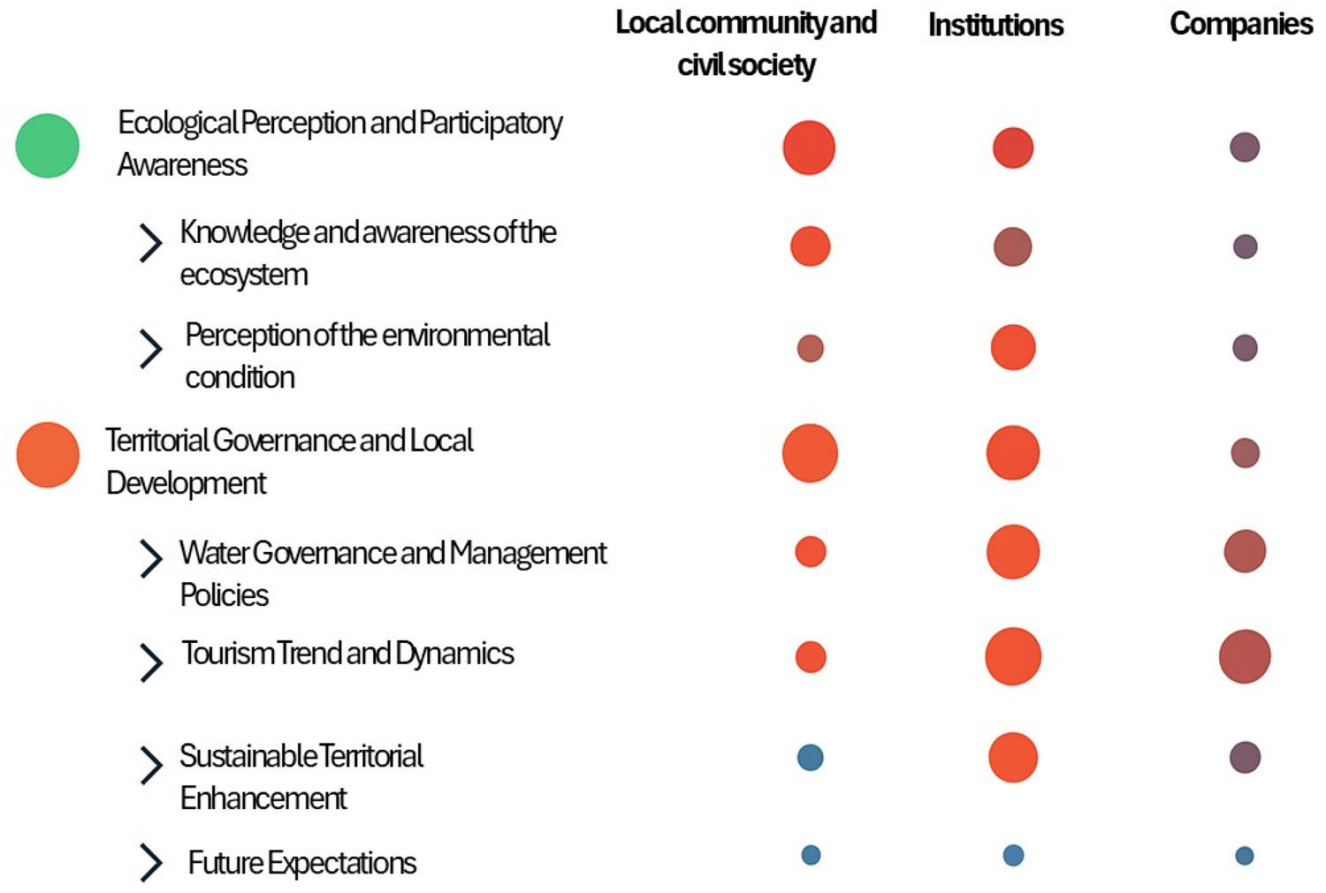
The matrix illustrates the frequency of the different qualitative codes across the three main stakeholder groups, thus the relative emphasis. The size of the dots corresponds to the relative frequency of the codes within each stakeholder category

Figure 4 provides a visual synthesis of the thematic relationships between stakeholder groups and the second order themes in the two aggregate dimensions. The results show that citizens and institutional actors are primarily engaged with themes related to Ecological Perception and Participatory Awareness, particularly concerning knowledge of the ecosystem and awareness of environmental change.

Conversely, tourism and accommodation businesses show a more concentrated association with themes under Territorial Governance and Local Development, where especially tourism trends and dynamics but also sustainable territorial enhancement appear as relevant. Following, water governance and management policies emerge as a shared concern, confirming that hydrological regulation and resource competition represent the intersection between ecological sustainability and socio-economic interests.

The following sections describe these two aggregate dimensions and their underlying second-order themes, supported by interview quotations and quantitative insights. Citation frequencies for each first-order concept are included to indicate which ideas were most commonly discussed, along with the percentage of interviewees who referred to each concept at least once during their interview.

### Ecological Perception and Participatory Awareness

This section examines in greater depth the findings related to *Ecological Perception and Participatory Awareness*, discussing the second-order themes that constitute this dimension. The results are presented alongside accompanying figures, which help identify the most salient and widely recognized concepts among respondents within each theme.

#### Knowledge and awareness of the ecosystem

**Fig. 5:**
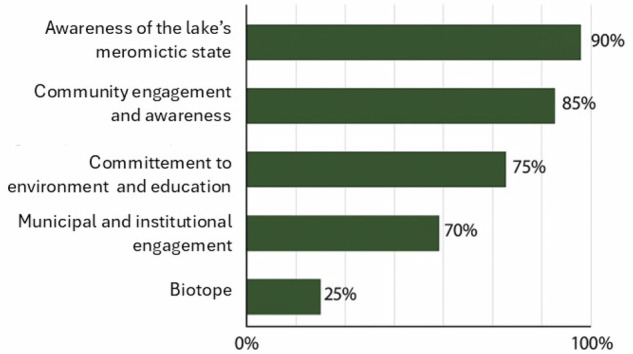
Percentage of interviewees mentioning each single first order concept in Knowledge and Awareness of the Ecosystem second order theme

The interview data reveal that local stakeholders demonstrate a strong attachment to the lake and regard it as essential to both the environmental and socio-economic well-being of the area. This emotional and practical connection often translates into an active interest in protecting the lake and acquiring both formal and informal knowledge about its ecological characteristics (Fig. 5).

A recurring theme is the awareness of the lake’s meromictic state, which emerged in the vast majority of interviews. While this topic was sometimes prompted by direct questioning, only about 10% of participants showed no knowledge of the phenomenon, although not all remaining respondents demonstrated a fully accurate understanding of it.

*“Let’s say I know a little about the meromixis, mainly through friends who work in the field. I’m not deeply informed, but I do know there are issues, like the increasingly thin layer of clean water. I’m aware there’s a problem”.* [#9] *“I’m not exactly sure what is meant by lake stratification. I know that part of the lake is considered ‘dead water’ because it’s stagnant, being meromictic. I think the lake is about 120 meters deep, maybe 35 meters are stagnant, and the rest is active… or maybe the other way around. I’m not sure”.* [#18]

The concept of community engagement and awareness was mentioned in 85% of the interviews, highlighting the perceived importance of public participation and concern for the lake. Several interviewees noted generational differences, with younger residents often perceived as less engaged.

*“There is a certain level of awareness, especially among older people, those who have witnessed the changes. For instance, when we ask for support from institutions during public hearings, it’s not hard to make people over 65 understand the severity of the situation. We’ve lived through it. […] Older residents understand the danger, not necessarily the stratification, but the damage caused by extreme water-level fluctuations. That’s something you can see”.* [#1]

The theme of commitment to environmental education and awareness-raising was also frequently discussed (75%), suggesting a shared recognition of the need to inform and involve the broader public. Many participants also emphasized the role of municipal institutions, at times explicitly calling for more proactive engagement. The Municipality of Idro was consistently cited as the most active and responsive actor, likely due to its larger size and direct involvement with the lakefront area.

*“The Municipality of Idro is the most involved and sensitive to these issues among the four. The other three tend to stay on the sidelines, maintaining a position of apparent neutrality that reveals an unwillingness to oppose the implementation of these interventions”.* [#14]

In contrast, the biotope, despite its designation as a Site of Community Importance (SCI), was rarely mentioned. This indicates a relatively low level of awareness or perceived relevance among respondents, even though lake interventions could directly affect it.

#### Perception of the lake’s environmental condition

Stakeholders’ perceptions of the lake’s environmental condition reveal a strong collective memory of the negative effects caused by the historically wide fluctuations in water levels, up to 7 m until 1987 and 3.25 m before 2008. Since 2008, the lake fluctuation has dropped to 1.30 m.

**Fig. 6:**
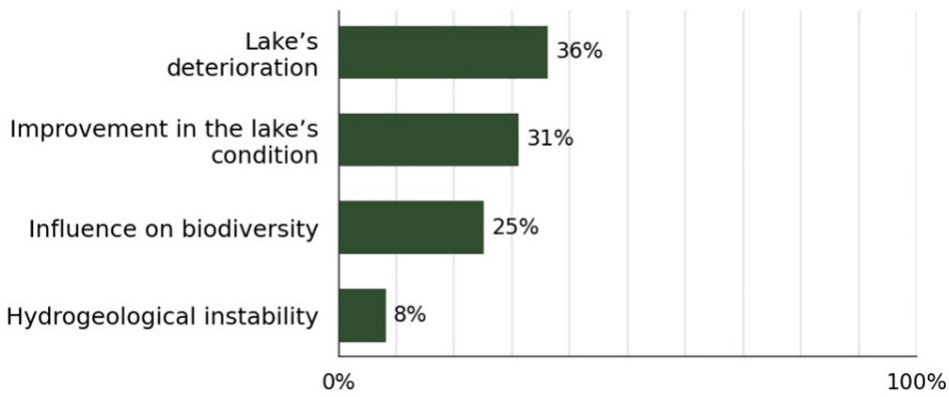
Percentage of respondents who mentioned each individual first-order concept in Perceptions of the Lake’s Environmental Condition’s second order theme

Many interviewees associated these large fluctuations with a general degradation of lake quality (Fig. 6), mentioning unpleasant visual and olfactory impacts such as algal blooms, foul odors, and negative effects on biodiversity.

The majority of respondents (81%) described a clear improvement in the lake’s condition following the more recent regulation of water levels to a maximum fluctuation of 1.30 m. This change is widely perceived as contributing to a healthier lake environment, both ecologically and esthetically. References to lake deterioration were frequent, with respondents emphasizing that the lake’s poorest conditions were observed during periods of greater water-level variation. Although some interviewees still noted signs of degradation, these were fewer and generally linked to occasional algal presence.

*“The main issue with Lake Idro right now is algae, really, it’s the algae. […] I went down to the shore and saw this greenish-blue foam. […] It turned out to be an algal bloom”. [#4]*

Another recurring theme (mentioned by 25% of respondents) concerned the influence on biodiversity, particularly with regard to fish populations and the disappearance of certain species such as the native marbled trout.

*“There used to be a native marbled trout that had found, in the area where the river widened into the lake, a perfect place to grow. […] But with the strong water-level fluctuations, fish eggs would be left exposed and dry. […] The trout that used to live permanently in the lake began to disappear because of those fluctuations”. [#10]*

Mentions of birdlife and aquatic vegetation were also present, though less frequent. In contrast, the issue of hydrogeological instability was rarely discussed.

### Territorial Governance and Local Development

This section examines in greater depth the findings related to *Territorial Governance and Local Development*, discussing the second-order themes that constitute this aggregate dimension.

#### Water governance and management policies

**Fig. 7:**
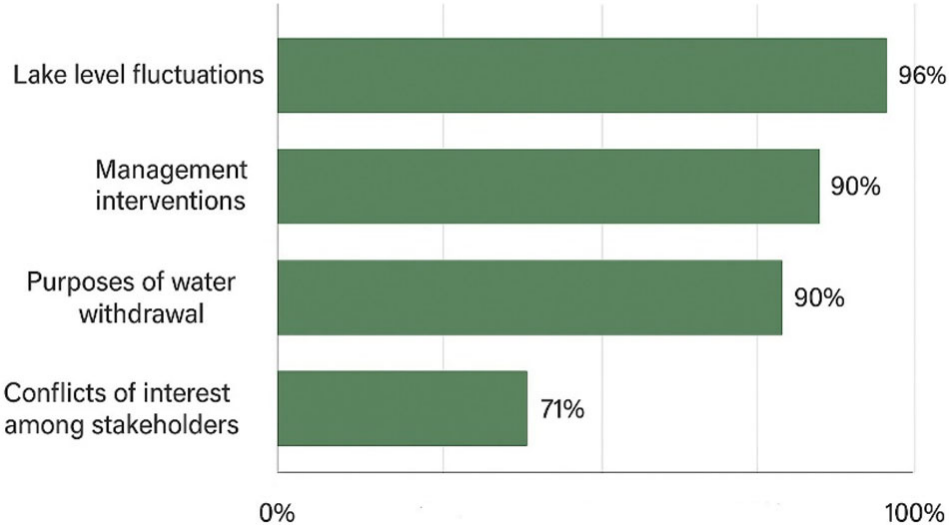
Percentage of interviewees mentioning each single first order concept in Water Governance and Management Policies second order theme

The governance of Lake Idro and the water management policies implemented over time are perceived by local stakeholders as highly significant (Fig. 7).

A central topic, mentioned in 96% of interviews, was the regulation of lake-level fluctuations, consistently linked to both ecological and esthetic transformations of the lake. At present, the regulated variation is limited to ~1.30 m, yet concern persists. This concern often translates into discussions about management interventions, such as the construction of water-collection tunnels and upgrades to the sewer system.

The purposes of water withdrawals, primarily for agriculture and hydroelectric production, are frequently debated. While some stakeholders expressed understanding of irrigation needs, they emphasized that withdrawals should remain within sustainable limits:

*“I’m not selfish, I need water for the lake and for tourism, of course, but I also understand the needs of agriculture, especially during severe droughts. […] If the lake is full and it drops by one vertical meter during the 15 to 20 days of peak irrigation in July and August, it’s not something that would really compromise the lake’s ecosystem”.* [#11]

However, skepticism was widespread regarding the official justification for upcoming infrastructure projects promoted by the Lombardy Region, specifically a third tunnel allegedly intended to improve hydraulic safety, but which many interpret as a pretext to increase water withdrawals:

*“These are poorly conceived projects that aren’t really about safety. The real aim is to lower the lake level by three and a half meters to meet the needs of intensive corn agriculture that still uses outdated irrigation methods”.* [#2]

In 71% of interviews, this issue evolved into a discussion of conflicts of interest among stakeholders. On one side, farmers and power companies benefit from water withdrawals; on the other, residents and business owners depend on the lake’s ecological stability and esthetic appeal to sustain the tourism-based economy:

*“The entire Chiese river system is exploited […] and the lake has suffered the most. […] It was historically treated as a reservoir for irrigation and energy. But now there’s tourism too, and that should be considered […]”.* [#20]

Some respondents also criticized the inefficiency of traditional irrigation technologies, such as open-channel flood irrigation, which they viewed as wasteful. Several suggested that modernization could reduce water demand and mitigate conflict, implicitly criticizing authorities for lacking the will to invest in more sustainable practices:

*“There’s a big difference between the agricultural areas in the provinces of Mantua and Cremona and those in Brescia. […] Brescia still relies heavily on flood irrigation, which consumes a huge amount of water. […] It shows a completely different approach to water as a resource”.* [#7]

#### Tourism trends and dynamics

Tourism emerges as the primary economic driver for the Lake Idro region, with both local authorities and hospitality operators emphasizing the importance of maintaining a lake that appears clean and ecologically healthy. Esthetic quality and environmental condition are perceived as directly linked to the area’s attractiveness to visitors.

Most interviewees reported that tourism has increased significantly in recent years, in parallel with the visible improvement of the lake’s environmental condition.

*“There has been an increase in tourist numbers because people have really started to appreciate Lake Idro. In the past, it was seen as an unknown place, even called the dark lake, almost just a basin. But recently, more people from nearby areas are coming, rediscovering the lake, also because it is now much cleaner than before”.* [#15]

**Fig. 8:**
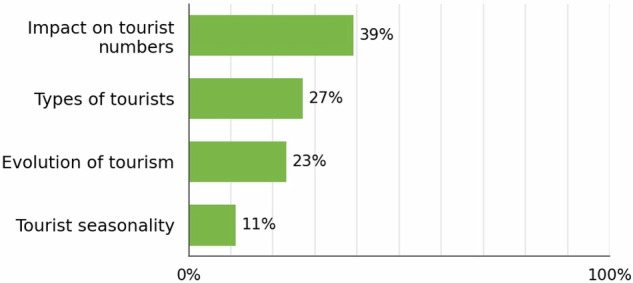
Percentage of respondents who mentioned each individual first-order concept in Tourism Trends and Dynamics’ second order theme

Many stakeholders linked this positive trend not only to ecological recovery but also to territorial improvements (Fig. 8), such as the development of cycling paths, outdoor recreation, and tourism infrastructure, as well as the growing accessibility offered by digital platforms like Airbnb.

*“With the rise of the internet and platforms like Airbnb, we’ve had visitors from everywhere. […] People might be looking at Lake Garda, then zoom out on the map, discover Lake Idro, see it’s cheaper, read good reviews, and think, ‘Well, this place looks nice too, let’s try it”.* [#17]

Nearly all participants reflected on how changes in the lake’s ecosystem have influenced visitor flows, observing that improved water quality has enhanced the lake’s reputation as a recreational destination.

About 76% of respondents, particularly institutional representatives and accommodation providers, discussed the evolution of tourism over time, providing insights into visitor numbers and profiles.

*“The main market is definitely German-Dutch, but in the last three or four years, since COVID, we’ve seen an increase in Italian visitors. […] Many of them tend to stay for short holidays, usually a weekend or four to five days. […] Lake Idro is still relatively uncrowded, and this has helped us as tourism operators increase both arrivals and overnight stays”.* [#16]

Although fewer interviewees (48%) explicitly mentioned seasonality, those who did consistently identified July and August as the peak tourist months. Some respondents also perceived a gradual expansion of the tourist season, driven by changing climatic conditions. This perception aligns with Matei et al. (2023), who project that climate change will alter traditional seasonal patterns of tourism demand across Europe. Specifically, northern regions are expected to see rising visitor numbers during summer and early autumn, while southern destinations may experience a relative decline in peak-season demand, partially offset by increased spring and autumn tourism.

#### Sustainable territorial enhancement

The theme of Sustainable Territorial Enhancement emerged as a particularly relevant topic across the dataset.

**Fig. 9:**
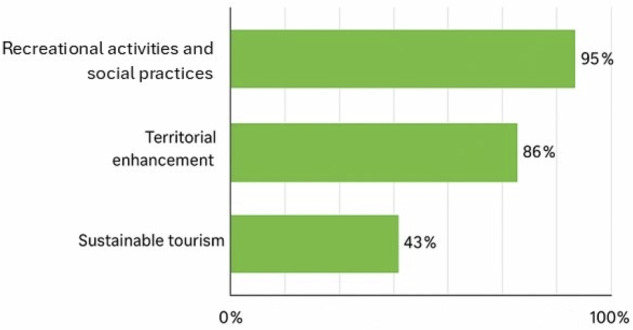
Percentage of interviewees mentioning each first-order concept in the Sustainable Territorial Enhancement’s second order theme

A large majority of participants (95%) referred to the evolution and diversification of recreational activities and social practices, emphasizing how leisure opportunities around the lake have expanded over time (Fig. 9). Traditional activities such as professional fishing, once a key source of income for the local population, have declined, while other sports, such as sailing, canoeing, windsurfing, kitesurfing, and diving, have become more common. Several interviewees also highlighted the recent growth of sport fishing, including carp fishing, marking both a cultural and ecological shift.

*“Fishing has changed, both professionally and recreationally. The professional fishing trade has lost importance, partly due to social changes. […] What’s really shifted is sport fishing: certain species, like pike, have declined, making room for others more suitable for different fishing techniques. […] This has led to an increase in methods like carp fishing”.* [#5]

Many respondents emphasized that recreational opportunities extend beyond the lake itself. Thanks to the surrounding mountainous landscape, activities such as hiking, cycling, and paragliding have become increasingly popular, contributing to the territory’s multifaceted appeal.

*“Our territory is becoming a suitable place for outdoor sports, including specific paragliding training. Schools from across Europe come here to run safety courses: they launch from the mountains above Bondone and perform specific maneuvers over the lake, with instructors providing guidance from boats. The lake allows them to train safely, as it serves as a safety buffer in case of emergency”.* [#8]

A high proportion of respondents (86%) also referred to territorial enhancement initiatives, including recent infrastructural developments aimed at increasing the area’s touristic attractiveness and supporting the diversification of recreational activities. One of the most frequently mentioned examples was the construction of a lakeside cycling path connecting Anfo to Ponte Caffaro, with plans to expand it to include other municipalities around the lake.

*“I believe the lake has a lot of potential that should be enhanced. The new cycle path will be a great asset for tourism, especially because the Lake Idro area has no factories, no resources—it must rely on nature and tourism. […] The current track connects Anfo and Bagolino, but the project also includes a link from Anfo to Idro. Ideally, we could have a full loop around the lake, but that’s complicated by the Trentino–Lombardy border”.* [#21]

Finally, some participants voiced suspicion about the regional government’s financial role. While many enhancement projects were funded by Regione Lombardia, a few respondents perceived this financial support as a form of political leverage, suggesting that regional funds were conditional upon local acceptance of projects that could increase water extraction and lake-level fluctuations. This perception reflects a persistent sense of conflict regarding the lake’s use and management.

*“The other municipalities around the lake are, unfortunately, somewhat blackmailed by having accepted this program agreement, because they took the money and carried out compensatory works”.* [#7]

In contrast, the topic of sustainable tourism was mentioned less frequently and, in less detail, suggesting that while infrastructural and recreational developments are valued, explicit strategies for promoting long-term sustainability in tourism remain less prominent in local discourse.

#### Future expectations

The analysis of stakeholder narratives revealed that reflections on the sustainability of lake management were central to understanding participants’ future concerns and aspirations.

**Fig. 10:**
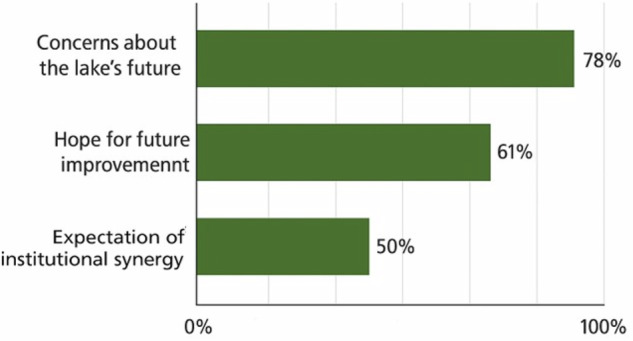
Percentage of interviewees mentioning each first-order concept in the Future Expectations’ second order theme

A prominent theme emerging from the interviews was widespread concern about the lake’s future condition (Fig. 10), particularly in relation to the potential implementation of the new regional project promoted by the Lombardy Region. This project, which includes the construction of a third tunnel, would increase water-level fluctuations to ~3.25 m. Many interviewees associated this scenario with the risk of ecological and esthetic deterioration, reminiscent of past periods when the lake’s condition was perceived as significantly worse.

These concerns were closely tied to expectations of negative socio-economic impacts, particularly on the tourism sector, which depends heavily on the lake’s environmental quality. Participants frequently noted that today’s visitors would be far less tolerant of degraded conditions than in the past, when environmental impacts were less visible and more readily accepted.

*“Now that I’ve been running my business for two years and started planning long-term, I’m growing increasingly concerned. If the new projects go forward, I’m certain tourist flows will drop significantly […] and there would be consequences for our business and investments. I can’t even fool myself into thinking that today’s visitors would tolerate the lake conditions of the 1970s–1980s, when there were strong fluctuations but people still came. […] I’m not optimistic at all”.* [#14]

While these fears dominated the discourse, 61% of participants expressed more cautious or optimistic views, often emphasizing uncertainty about whether the new project would actually proceed.

*“If things remained as they are now, it would be perfect, water levels are ideal, and the lake is very clean. We’ll see how things will be affected in the future. […] If legislation and current agreements stayed the same, everything would be fine. Let’s really hope they don’t change with these new interventions”.* [#15]

Approximately half of the interviewees also reflected on the importance of institutional collaboration (*Expectation of Institutional Synergy*) as a key factor in ensuring a stable or improved future scenario. Some participants hoped for stronger cooperation among local municipalities to oppose regional plans with a unified position, while others advocated for constructive dialogue between local and regional authorities to identify balanced, sustainable compromises.

*“Working in synergy pays off. If there’s no cooperation, you’re doomed. […] When we create something, we need to coordinate, I do a part, then you add your piece, then another municipality adds theirs, and from there, great things can happen. Small communities collaborating can achieve a lot. […] In the past, there was a lot of parochialism, but now we need to broaden our horizons. The new bike path is an example”.* [#11]

*“I don’t see much chance for a thaw between the two sides, there’s a clear communication gap. One side doesn’t want to listen, and the other refuses to compromise. There’s this duality: one wants to go ahead no matter what, and the other says those plans must not happen. […] There’s maybe a flicker of hope, but I think it’s tough. The community has valid concerns, but civil coexistence requires some give and take, proposals and counterproposals need to be fairly assessed”.* [#5]

Overall, concern about future deterioration emerged as the most frequently coded concept within this theme, particularly regarding the potential for increased water-level fluctuations. By contrast, hopes for future improvement were mentioned less often, perhaps because they represented a more abstract aspiration and were usually expressed only once per interview. In contrast, fears of deterioration were rooted in direct past experience, making them more concrete and frequently revisited throughout the interviews.

## Discussion

Although grounded in the specific context of Lake Idro, the findings of this study reflect dynamics that are increasingly common across regulated alpine and peri-alpine lake systems. Many deep subalpine lakes in Europe share a history of intensive water exploitation for hydropower and agriculture, followed by a gradual reorientation toward environmental protection, recreation and tourism. Similar dynamics have been observed in regulated subalpine lake systems, where competing water uses, changing management priorities, and the gradual institutionalization of environmental concerns shape long-term sustainability trajectories (Casale et al. 2021; Maran et al. 2023).

The Lake Idro case illustrates how relatively small adjustments in water-level management, such as the reduction of fluctuation amplitude, can generate perceptible improvements in surface ecological conditions that are readily recognized by local stakeholders. These perceptions are broadly consistent with scientific evidence documenting improved surface-water quality despite the persistence of deep-water anoxia and stable meromixis (Tartari et al. 2021). At the same time, recent basin-scale analyses of deep subalpine lakes indicate that external nutrient pressures remain substantial and, in several catchments, are declining only slowly (Dezuanni et al. 2025). This coexistence of visible recovery and latent vulnerability highlights a structural feature of regulated lake systems: ecological improvements are often partial, spatially stratified and uneven across ecosystem compartments (Søndergaard et al. 2007; Wantzen et al. 2008, Zohary and Ostrovsky 2011; Rogora et al. 2018).

A key contribution of this study lies in showing how environmental change becomes socially meaningful through processes of visibility and interpretation. Stakeholders’ narratives reveal that perceptions of sustainability are strongly anchored in observable phenomena, such as water clarity, shoreline stability, or algal presence, while less visible processes, including meromixis, hypolimnetic anoxia, or manganese-mediated biogeochemical dynamics, remain marginal in public discourse.

This pattern closely aligns with the concept of territorialization of environmental concerns, which describes how environmental issues gain political relevance when they become visible, measurable, and narratively framed within specific governance arenas (Maran et al. 2023). In Lake Idro, water-level fluctuations act as a powerful territorializing mechanism: they translate complex hydrological and ecological processes into tangible, experiential signals that structure local understandings of sustainability. Past episodes of severe fluctuation function as collective reference points, shaping current evaluations of ecological improvement and amplifying fears of future regression (Coleman et al. 2016; Vogelpohl et al. 2025).

Conversely, processes that remain largely invisible, such as the stable stratification of the water column and the deep turbid stratum described by limnological research, are weakly integrated into local sustainability narratives, despite their ecological significance (Tartari et al. 2021).

Agriculture also deserves specific attention in the Lake Idro case, as irrigation demand is one of the main drivers of water-level regulation and stakeholder conflict. Interviewees repeatedly associated past and potential future fluctuations with downstream agricultural withdrawals, especially during summer drought periods and questioned whether current irrigation practices are compatible with ecological and tourism-related objectives. This highlights the need to interpret Lake Idro as a cross-sectoral water-governance system, in which agriculture, hydropower, tourism and environmental protection are interdependent rather than separate policy domains. From an adaptive water management perspective, addressing these tensions requires coordinating demand-side measures, irrigation efficiency and transparent negotiation among upstream and downstream water users (Casale et al. 2021; Maran et al. 2023; Pahl-Wostl 2009).

The findings further demonstrate that local perceptions act as mediating mechanisms between ecological change, institutional trust and governance outcomes. Stakeholders’ narratives reveal not only how environmental change is interpreted, but also how these interpretations shape confidence in public authorities and management decisions. In Lake Idro, distrust toward regional institutions emerges as a recurring theme, particularly in relation to large-scale infrastructural projects perceived as externally imposed. This pattern is consistent with recent evidence showing that distrust of government is widespread in lake governance contexts, especially where local communities are deeply attached to the resource and highly engaged in its stewardship. Zhang et al. (2024) show that public stakeholders and local residents tend to be strongly involved and passionate about issues concerning their local lakes, while simultaneously expressing skepticism toward governmental decision-making processes when these are perceived as opaque, top-down, or misaligned with local priorities. This combination of high local involvement and low institutional trust represents a critical challenge for environmental management. As also highlighted by Maran et al. (2023), water management arenas are shaped by power relations and contested forms of visibility, in which environmental concerns gain legitimacy only when they are recognized as relevant and actionable within institutional frameworks.

The analysis also reveals a persistent gap between scientific knowledge and public understanding of lake ecosystems. While stakeholders demonstrated awareness of general ecological improvement, their limited engagement with less visible processes mirrors findings from limnological research showing that deep subalpine lakes can exhibit surface recovery alongside ongoing internal biogeochemical instability (Tartari et al. 2021). Moreover, recent evidence on nutrient loading suggests that catchment-scale pressures remain a critical driver of long-term lake dynamics, even where local surface conditions appear improved (Dezuanni et al. 2025). From an environmental management perspective, this gap does not simply call for more information provision but for processes of knowledge exchange and co-production that can make scientific evidence salient, credible and legitimate for local actors and decision-makers. In complex socio-ecological systems, scientific knowledge is more likely to inform management when it is connected to stakeholders’ concerns, institutional contexts and lived experience, rather than communicated as a one-way transfer of expert information. Enhancing ecological literacy therefore requires participatory forms of knowledge exchange that frame complex processes in ways that are meaningful for those involved in lake governance. At the same time, knowledge exchange should not be assumed to produce automatic or immediate outcomes (Cash et al. 2003; Norström et al. 2020). Tourism emerges in this study as both a beneficiary of ecological improvement and a potential source of new pressures, such as intensified recreational use, increased accessibility and the seasonal concentration of visitors in ecologically sensitive areas. Stakeholders consistently linked improved environmental conditions to increased tourist flows, reinforcing the role of lakes as providers of cultural and recreational ecosystem services. This perception is consistent with broader research showing that mountain lakes are valued primarily for passive recreation, esthetic quality, and nature-based experiences (Schirpke et al. 2021).

At the same time, the findings reveal an implicit tension between territorial enhancement and long-term sustainability. While infrastructural developments and improved accessibility are widely welcomed, explicit strategies for managing visitor pressure and ecosystem limits remain underdeveloped. Schirpke et al. (2021) demonstrate that increasing accessibility can shift visitor profiles and intensify conflicts between nature-oriented and leisure-oriented users, with potential consequences for ecological integrity and perceived environmental quality.

In Lake Idro, similar dynamics are emerging, even if not yet fully articulated in local discourse. This suggests that tourism-related sustainability challenges in alpine lake systems are not primarily driven by visitor numbers alone, but by governance capacity to anticipate trade-offs between accessibility, ecosystem protection and social expectations.

Beyond its empirical findings, this study offers a methodological contribution by demonstrating the value of integrating qualitative, perception-based approaches with ecological evidence in environmental management research. While limnological studies provide essential insights into biogeochemical processes and nutrient dynamics (Tartari et al. 2021; Dezuanni et al. 2025), perception-based analyses reveal how these processes are interpreted, contested, or ignored within governance arenas.

This integrative approach is particularly relevant in contexts characterized by slow-onset environmental change where conventional indicators may fail to capture emerging social tensions or shifts in legitimacy. By situating stakeholder perceptions alongside scientific knowledge, environmental managers can better anticipate conflicts, design adaptive interventions, and align policy objectives with socially grounded understandings of sustainability.

## Conclusions

This study explored how local stakeholders perceive the sustainability of ecological and socio-economic changes affecting Lake Idro, a complex alpine water system at the intersection of environmental management, tourism, and regional governance. This study examined how stakeholders perceive ecological change and sustainability in a regulated deep lake system, using Lake Idro as an illustrative case. The findings show that sustainability is not understood solely through biophysical indicators, but emerges from the interaction between visible ecological change, lived experience, governance arrangements, and institutional trust. Stakeholder perceptions of improvement following reduced water-level fluctuations coexist with persistent concerns about future interventions, highlighting how sustainability assessments are shaped as much by social interpretation and memory as by ecological conditions.

The results underscore that environmental management in regulated lake systems is inherently socio-ecological. Perceptions of legitimacy, trust in institutions, and the visibility of environmental change play a central role in shaping acceptance of management measures and willingness to support long-term strategies. Where ecological improvements are visible but underlying vulnerabilities remain less perceptible, there is a risk that management success is evaluated prematurely, potentially weakening support for preventive or adaptive interventions. Addressing this gap requires not only technical monitoring, but also governance approaches capable of translating complex ecological processes into socially meaningful knowledge. From a management perspective, cross-sectoral coordination among water management, agriculture, tourism, and environmental protection is essential to reconcile competing resource uses and to address historical governance lock-ins. The Lake Idro case shows that long-term sustainability cannot be pursued through technical regulation alone. It requires iterative management processes that combine ecological monitoring, stakeholder knowledge, transparent decision-making, and the capacity to adjust rules and infrastructure in response to changing environmental conditions and social expectations. In regulated lake systems characterized by slow ecological responses and delayed feedbacks, adaptive management can help bridge the gap between biophysical evidence and locally perceived sustainability, while strengthening the legitimacy of future interventions (Pahl-Wostl et al. 2007; Huitema et al. 2009; Pahl-Wostl 2009). Future research should expand the spatial scope to include other subalpine lakes, which could help identify broader patterns and governance models applicable at a regional scale. Furthermore, longitudinal research tracking changes in perception before and after the implementation of new infrastructure projects would provide valuable insights into how policy decisions reshape public trust and environmental attitudes.

Finally, this study acknowledges limitations. The relatively small, purposive sample, while offering depth of understanding, limits generalizability to the broader population. In addition, the focus on local stakeholders may underrepresent the perspectives of regional policymakers and agricultural water users, whose inclusion could deepen the understanding of cross-scale governance tensions.

Overall, the study contributes to ongoing debates on multi-level water governance and sustainability transitions in alpine regions. By foregrounding local perceptions, it reveals both the progress and the fragility of lake’s environmental recovery and underscores the importance of participatory, evidence-informed policymaking in managing natural resources amid competing socio-economic interests.

## Data Availability

The data analyzed during the SEBINO project (project code: 2022E9EZHN) are available from the corresponding author upon reasonable request.
